# Amo—I Love

**Published:** 1899-09

**Authors:** 


### "AMO—*I LOVE.*"

OWING to the "unparliamentary" language used against George M. Gould, of the *Philadelphia Medical Journal* (a doodle-bug that will come to the surface only when the straw is baited with the subscription price) by I. N. Love, of the *St. Louis Medical Mirror*, from which John T. Milliken has recently scraped various irregular fragments of quicksilver, leaving a rather distorted and refracted image of the editor, the medical journalists are calling for his resignation as one of the members of the A. M. A.

Love is a well-known puffer of proprietary compounds, almost equalling the hyphenated dermatologist of the same city, and that fact alone ought to render his seat among the mighty an uneasy one.

In the present difficulty, if name is indicative of character, Love should rush into Gould's arms and let the scalding tear of repentance trickle down his massive torso.

---

In the last issue of the JOURNAL a serious error was committed by the printer mixing together two distinct book reviews. On page 393, line 19, under the review of Professor Fuchs' work on Ophthalmology, a portion of the review of Dr. Hollopeter's little book on Hay-Fever was inadvertently placed. This, of course, makes exceedingly ridiculous reading. Line 19 on page 393 should be placed at the end of the paragraph on page 395 under "Hay-Fever and Its Successful Treatment." One who knew the work of Professor Fuchs would certainly know also that such criticisms as were there found could not be applicable to his book; and yet it is for those who are not so familiar that attention is drawn to this error.

---

THE AMERICAN ELECTRO-THERAPEUTIC ASSOCIATION.—With the near approach of the annual meeting of the American Electro-Therapeutic Association, to be held on September 19th, 20th and 21st, in the city of Washington, under the presidency of Dr. F. B. Bishop, the local committee of arrangements is redoubling its efforts to make it a success. Many electrical manufacturers will be represented in the exhibit hall, and information will be freely given. Papers have thus far been promised from Drs. R. G. Nunn, A. D. Rockwell, Margaret A. Cleaves, F. J. Leviseur, Walter White, Robert Reyburn, G. A. Corson, C. O. Files, J. H. Kellogg, John A. Licthy, W. W. Scheppegrell, L. Howe, E. Wende, F. B. Bishop, Robert Newman, W. J. Herdman, G. B. Massey, and Professors Bergonia of Bordeaux, Apostoli and Dolbear of Paris. With such authors, a successful meeting must result.

